# Atypical Arytenoid Lesion Revealing Lobular Capillary Hemangioma: Diagnostic and Therapeutic Challenges

**DOI:** 10.7759/cureus.109347

**Published:** 2026-05-21

**Authors:** José Gonzalo Bravo Quiroz, Ana G Saavedra Mendoza, José Manuel García Romero, Hillary Lizarraga Payan, José Carlos Acosta Careaga

**Affiliations:** 1 Otolaryngology-Head and Neck Surgery, Hospital General Dr. Manuel Gea González, Mexico City, MEX; 2 Plastic Surgery, University Hospital Coventry and Warwickshire, Coventry, GBR; 3 Pathology, Hospital General Dr. Manuel Gea González, Mexico City, MEX

**Keywords:** arytenoid, blue laser, contact granuloma, larynx, lobular capillary hemangioma, pyogenic granuloma

## Abstract

Lobular capillary hemangioma (LCH), historically referred to as pyogenic granuloma, is a benign vascular proliferation rarely identified within the larynx. Due to its uncommon location and variable endoscopic appearance, laryngeal LCH may mimic other benign laryngeal lesions, including contact granuloma, creating diagnostic and therapeutic challenges. Unlike conventional laryngeal granuloma, which is frequently managed conservatively, LCH often requires surgical excision and histopathological confirmation for definitive diagnosis.

We present the case of an 18-year-old female patient with persistent globus sensation, dysphonia, and odynophagia who was found to have an atypical exophytic lesion arising from the right arytenoid cartilage. Initial endoscopic evaluation demonstrated a smooth, translucent, well-circumscribed lesion without overt ulceration or typical inflammatory features of contact granuloma. Given the atypical appearance of the lesion, persistent symptomatology, and ongoing diagnostic uncertainty, direct laryngoscopy with blue laser excision was performed for both diagnostic and therapeutic purposes. Histopathological examination revealed LCH. Follow-up at three and eight weeks demonstrated complete symptomatic resolution without evidence of recurrence.

This case highlights the importance of considering LCH within the differential diagnosis of atypical laryngeal granulomatous lesions. Histopathological evaluation remains essential in lesions with unusual endoscopic characteristics, as management strategies and therapeutic implications may differ significantly from those of conventional laryngeal granuloma.

## Introduction

Laryngeal granuloma, also referred to as contact granuloma or vocal process granuloma, represents a relatively common benign inflammatory lesion frequently associated with laryngopharyngeal reflux, vocal abuse, chronic cough, endotracheal intubation, or repetitive mechanical trauma. These lesions are classically located on the vocal process of the arytenoid cartilage and are commonly managed with conservative treatment, including proton pump inhibitors, voice therapy, inhaled corticosteroids, and elimination of contributing factors [1,2]. Surgical intervention is generally reserved for refractory cases, airway compromise, or diagnostic uncertainty [2].

In contrast, lobular capillary hemangioma (LCH), historically termed pyogenic granuloma, is a benign vascular proliferation characterized histologically by lobular arrangements of capillary-sized vessels within an edematous and inflamed stroma [3,4]. Despite its traditional nomenclature, this lesion is neither pyogenic nor granulomatous [3,4]. LCH most frequently affects the skin and oral mucosa, while laryngeal involvement remains exceptionally uncommon [5,6].

Because of its rarity and nonspecific endoscopic appearance, LCH may clinically resemble other benign laryngeal lesions, particularly conventional laryngeal granuloma. This overlap may create significant diagnostic and therapeutic challenges, as management strategies differ considerably between these entities. While conventional contact granulomas are frequently approached conservatively, LCH often requires surgical excision and histopathological confirmation for definitive diagnosis.

We present the case of an atypical arytenoid lesion initially considered within the differential diagnosis of laryngeal granulomatous lesions, ultimately diagnosed as LCH following surgical excision and histopathological evaluation.

## Case presentation

An 18-year-old female patient presented with persistent globus sensation, dysphonia, and odynophagia of several weeks' duration. She had a recent history of accidental ingestion of a metallic thumbtack approximately five months before presentation, requiring urgent direct laryngoscopy and foreign body extraction from the epiglottis without complications. Following the procedure, the patient remained asymptomatic during subsequent follow-up visits.

Five months later, she developed persistent globus sensation, dysphonia, and odynophagia without dyspnea, stridor, or respiratory distress. Flexible laryngoscopic evaluation revealed an atypical exophytic lesion arising from the right arytenoid region. The lesion appeared smooth, translucent, well-circumscribed, and polypoid, without ulceration or the characteristic inflammatory appearance commonly associated with conventional contact granuloma. Vocal fold mobility was preserved bilaterally, and the glottic airway remained patent. 

Although the patient had a previous history of laryngeal foreign body extraction, the lesion's anatomical location and endoscopic appearance did not clearly support a direct causal relationship. Given the persistence of globus sensation, dysphonia, and odynophagia, in addition to the lesion's atypical endoscopic characteristics and diagnostic uncertainty, surgical excision was considered both diagnostically and therapeutically appropriate.

The patient underwent direct laryngoscopy with resection of the right arytenoid lesion using blue laser technology. The procedure was completed without complications, preserving surrounding laryngeal structures and vocal fold mobility.

**Figure 1:**
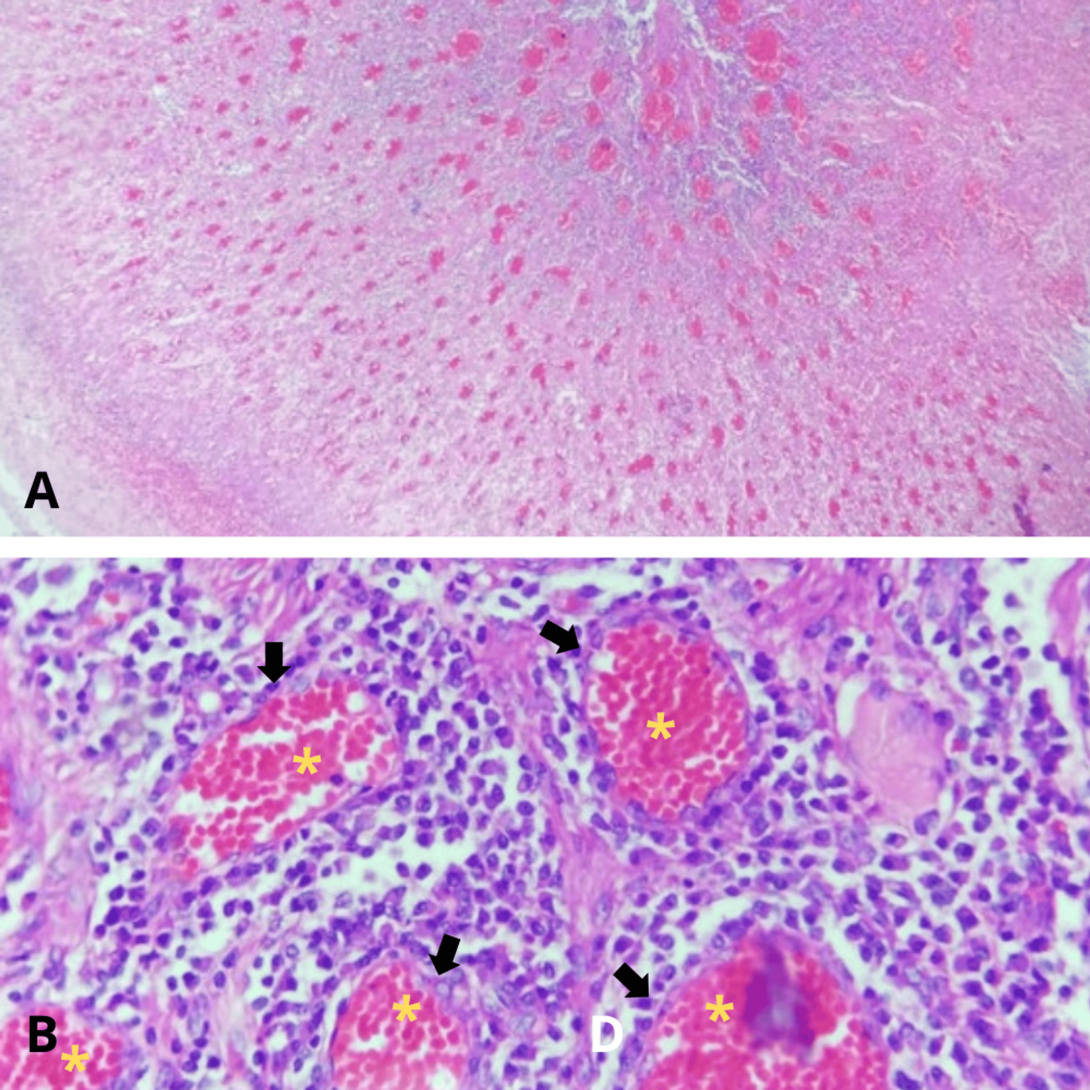
Histopathological findings (A) Low-power hematoxylin and eosin staining demonstrating lobular vascular proliferation with capillary-sized vessels and inflammatory stroma. (B) High-power hematoxylin and eosin staining demonstrating capillary vascular channels (black arrows) containing erythrocytes (yellow asterisks) within a lobular arrangement, consistent with lobular capillary hemangioma.

Histopathological examination demonstrated lobular proliferation of capillary-sized vascular channels within an inflamed stroma, consistent with LCH (Figure 1). 

**Figure 2:**
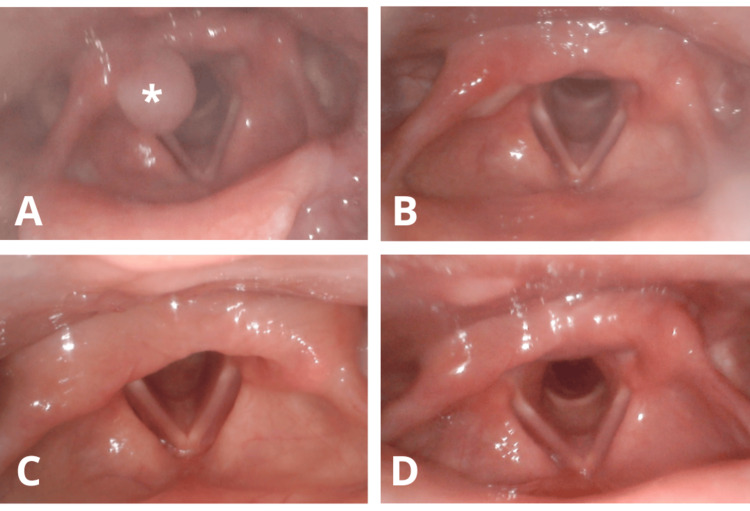
Flexible laryngoscopic evolution (A) Preoperative endoscopic evaluation demonstrating a smooth, translucent, exophytic lesion arising from the right arytenoid region (white asterisk) with preserved glottic airway. (B) Three weeks' follow-up after blue laser excision demonstrating adequate healing without evidence of residual lesion. (C) Eight weeks' follow-up showing adequate healing without evidence of residual lesion with preserved laryngeal anatomy and glottic function. (D) Four months' follow-up showing complete resolution of the lesion with preserved laryngeal anatomy and glottic function.

Postoperative follow-up at three weeks, eight weeks, and three months demonstrated complete symptomatic resolution with no evidence of recurrence on endoscopic examination. Adequate healing of the arytenoid region and preserved laryngeal function were observed (Figure 2). 

## Discussion

LCH is a benign vascular lesion most commonly encountered within the oral cavity, nasal mucosa, and skin [3,5]. Laryngeal involvement is exceedingly rare, with only isolated cases described in the literature [6,7]. The lesion has historically been referred to as pyogenic granuloma, although this terminology is considered a misnomer because the lesion demonstrates neither infectious etiology nor granulomatous inflammation histologically [3,4].

Conventional laryngeal granuloma represents a distinct pathological entity. Unlike LCH, contact granuloma is considered a reactive inflammatory lesion associated with repetitive mucosal trauma, laryngopharyngeal reflux, intubation injury, chronic throat clearing, or vocal abuse [1,2]. These lesions typically arise from the vocal process of the arytenoid cartilage and are frequently managed conservatively through antireflux therapy, voice therapy, inhaled corticosteroids, and modification of triggering factors [1,2].

Several studies and systematic reviews emphasize that surgical intervention for conventional laryngeal granuloma should generally be reserved for refractory disease, airway compromise, or situations in which diagnostic uncertainty persists [1,2]. This distinction becomes clinically relevant because atypical lesions may mimic contact granuloma endoscopically while representing entirely different pathological processes requiring alternative management. The key distinguishing features between these two entities are summarized in Table 1.

**Table 1:** Comparative features of lobular capillary hemangioma and contact granuloma of the larynx Table adapted from Mills et al. [3] and Chang et al. [2].

	Lobular capillary hemangioma	Contact granuloma
Definition	Benign vascular lesion characterized by lobular capillary proliferation	Benign reactive inflammatory lesion composed of chronic granulation tissue secondary to mechanical trauma or irritation
Anatomical location	Variable; may arise from any laryngeal mucosal surface, including the arytenoid, epiglottis, or subglottis; normally from the skin, oral mucosa, and tongue	Typically located on the vocal process of the arytenoid cartilage; bilateral involvement occurs in up to 20% of cases
Endoscopic appearance	Smooth, translucent, well-circumscribed polypoid lesion with prominent vascularity and easy bleeding	Sessile or pedunculated erythematous lesion, frequently ulcerated and covered by fibrinous inflammatory tissue
Histology	Lobular proliferation of capillary-sized vessels within the inflamed stroma without true granulomatous inflammation or necrosis	Granulation tissue with fibrinous exudate, chronic inflammatory infiltrate, and reactive squamous epithelium
Etiology	Usually idiopathic; associated factors include trauma, hormonal influences, medications, and occasionally infection	Commonly associated with laryngopharyngeal reflux, vocal abuse, endotracheal intubation, and repetitive mucosal trauma
Symptoms	Rapid growth and easy bleeding; respiratory symptoms are uncommon	Hoarseness, chronic cough, globus sensation, throat discomfort, and occasional dyspnea
First-line treatment	Surgical excision, preferably with laser techniques, followed by histopathological confirmation	Conservative management with proton pump inhibitors, voice therapy, inhaled corticosteroids, and removal of triggering factors
Role of surgery	Primary: excision is both diagnostic and therapeutic	Reserved for refractory disease, airway compromise, or diagnostic uncertainty
Recurrence risk	Low after complete excision; risk higher with incomplete resection	Moderate; dependent on the elimination of underlying causative factors
Rarity	Exceedingly rare at the laryngeal site	Common; among the most frequent benign laryngeal lesions

In the present case, the lesion demonstrated several atypical characteristics that raised diagnostic uncertainty. Although the lesion originated from the arytenoid region, its location along the superior aspect of the arytenoid cartilage was not characteristic of a conventional contact granuloma, which classically arises from the vocal process. In addition, the lesion lacked the ulcerative and inflammatory appearance typically associated with reactive laryngeal granuloma, instead appearing smooth, translucent, rounded, and exophytic. These atypical anatomical and endoscopic features significantly increased diagnostic uncertainty.

Although the patient had a history of prior direct laryngoscopy for foreign body extraction, the relationship between this procedure and the subsequent development of LCH warrants consideration. Traumatic or mechanical injury has been implicated as a precipitating factor for LCH at various mucosal sites, including the larynx [3,7]. In the present case, however, the lesion arose from the superior arytenoid surface rather than the vocal process or posterior commissure, regions most commonly subjected to intubation-related trauma, and developed approximately five months after the procedure, a longer interval than that typically observed in post-intubation granuloma formation. While a post-traumatic mechanism cannot be excluded and may have contributed to vascular proliferation, the atypical anatomical location and delayed onset suggest that additional or alternative etiological factors may have been operative.

The diagnostic dilemma represented one of the most important aspects of this case. Had the lesion been presumed to represent a typical contact granuloma, initial conservative management alone might have been selected. However, the persistence of globus sensation, dysphonia, and odynophagia, combined with the lesion's atypical endoscopic appearance, supported surgical intervention for both symptom resolution and definitive histopathological diagnosis.

Histopathological examination ultimately established the diagnosis of LCH, retrospectively confirming the appropriateness of surgical management. This finding significantly altered the interpretation of the lesion, emphasizing the limitations of endoscopic appearance alone in differentiating atypical laryngeal lesions [3,6].

Another relevant aspect of this case involves the use of blue laser technology for excision. Blue laser systems offer selective affinity for vascular lesions while preserving adjacent laryngeal structures, making them particularly useful for minimally invasive management of benign vascular lesions of the larynx [6]. In our patient, blue laser excision allowed complete resection with the preservation of laryngeal anatomy and excellent postoperative functional outcomes.

This case highlights the importance of maintaining a broad differential diagnosis when evaluating atypical arytenoid lesions. While conventional contact granuloma remains common, unusual endoscopic characteristics should prompt the consideration of alternative pathological entities, including LCH [6,7]. Histopathological analysis remains essential in cases with diagnostic uncertainty, as therapeutic implications may differ substantially [1,2,6].

## Conclusions

LCH of the larynx represents a rare benign vascular lesion that may clinically and endoscopically resemble a conventional laryngeal granuloma. However, atypical anatomical localization outside the classic vocal process region, together with unusual endoscopic characteristics and persistent symptomatology, should raise suspicion for alternative pathological entities. Distinguishing between these entities is clinically important because their therapeutic approaches differ significantly.

Longer follow-up, ideally at 12 months, would be valuable to confirm durable remission, as recurrence following incomplete excision has been described in LCH at other mucosal sites.

This case emphasizes that atypical laryngeal lesions with persistent symptomatology and unusual endoscopic characteristics may warrant histopathological evaluation, particularly when diagnostic uncertainty persists. Definitive histopathological diagnosis remains essential to guide appropriate management, achieve symptom resolution, and avoid potential therapeutic misclassification.
